# Expression and Transcriptional Regulation of Human *ATP6V1A* Gene in Gastric Cancers

**DOI:** 10.1038/s41598-017-03021-3

**Published:** 2017-06-07

**Authors:** Pin Wang, Lei Wang, Jie Sha, Guochun Lou, Nannan Lu, Bo Hang, Jian-Hua Mao, Xiaoping Zou

**Affiliations:** 1 Department of Gastroenterology, Drum Tower Clinical Medical School of Nanjing Medical University, Nanjing, Jiangsu 210008 China; 2 Biological Systems and Engineering Division, Lawrence Berkeley National Laboratory, Berkeley, CA 94720 USA; 3 Department of Gastroenterology, Jingjiang People’s Hospital, Jingjiang, Jiangsu 214500 China; 4 Department of Gastroenterology, Zhejiang Provincial People’s Hospital, Hangzhou, Zhejiang 310014 China; 5 Department of Oncology, The Affiliated Provincial Hospital, Anhui Medical University, Hefei, Anhui 230001 China

## Abstract

Recent studies demonstrate that the invasion and metastasis of gastric cancer (GC) is closely associated with a multi-subunit vacuolar H+-ATPase (V-ATPase). Here we investigated the expression and role of the human *ATP6V1A* gene that encodes the catalytic subunit A of V-ATPase in GC. We found that *ATP6V1A* expression level is significantly elevated in GCs compared to normals, but GC patients with higher expression levels of *ATP6V1A* have a better prognosis. Genomic analysis revealed that *APT6V1A* copy number is gained in a small fraction of GC patients and lost in a minimum number. Moreover, the *ATP6V1A* copy number was positively correlated with its mRNA level. To explore additional mechanisms by which *ATP6V1A* overexpressed in GCs, we investigated the relationship between transcription factor YY1 and ATP6V1A, and found that mRNA expression of *YY1* had significant correlation with that of *ATP6V1A*. To validate that YY1 transcriptionally regulates *ATP6V1A*, we discovered that the *ATP6V1A* core promoter region contains three YY1 binding sites. Moreover, RNAi-mediated knockdown of YY1 in GC cells significantly decreased *ATP6V1A* mRNA and protein expression, while YY1 overexpression increased *ATP6V1A* expression level. In conclusion, YY1 may play an important regulatory role in *ATP6V1A* expression with potential mechanistic and clinical implications in GC.

## Introduction

Gastric cancer (GC), as a malignant tumor originating from gastric mucosal epithelial cells, is the fourth most common cancer worldwide following lung cancer, breast cancer and colorectal cancer^1, 2^. GC is a multi-gene disease caused by the interaction of multiple cancer-promoting and suppressing genes with the microenvironment, leading to early pathological changes of the gastric mucosa followed by abnormal hyperplasia^3, 4^. Microarray and next generation sequencing (NGS) technologies have been invaluable tools to deconvolute the heterogeneity and complexity of somatic GC genetics, providing tremendous information to define new biomarkers for diagnosis, prognosis and prediction of therapeutic response, and to identify new potential therapeutic targets^5, 6^. However, while some improvements have been made in diagnosis and treatment of GC, the prognosis and survival for most patients, especially those with metastasis, have not dramatically changed^7^. Furthermore, to fulfill the promise of precision GC medicine, it is critical to understand the functional role and mechanism of these identified genomic changes in GC development and to explore them as potential therapeutic targets.

It has been shown in recent years that the invasion and metastasis of gastric cancer is closely associated with vacuolar H+-ATPases (V-ATPases)^8–10^. As a specific proton pump on the membrane of GC cells, the V-ATPases play an important role in the maintaining of a relatively neutral pH in normal cells and the acidification of the microenvironment in tumor^11, 12^. The latter is one of the most pronounced characteristics of tumor cells, and such acidic microenvironment strongly influences tumor progression^13^.

The V-ATPases is a complex multi-subunit transmembrane proton transport enzymes that widely exist in the cytoplasmic membrane of eukaryotic cells and the membrane system of cytonem^14–16^. In addition to its distribution on tumor cell membranes to maintain the tumor acidic microenvironment, a large number of V-ATPases are also present on the membrane of cytolysosomes and autolysosomes to maintain the intramembranous acidic environment with a pH of 5 required by hydrolases in these organelles. Through the regulation of the acidification of cytolysosomes, V-ATPases are involved in the degradation of proteins and their intracellular transport and sorting^17–19^. In tumor cells, in addition to the acidifying effect, the enhanced activity of V-ATPases and other proton transporters on the cell membrane is closely related to the proliferation of tumor cells and the migration of invasive cells^20^.

V-ATPases are composed of two structural domains, *i*.*e*., transmembrane V0 and intramembranous V1. The former provides a channel for H+, and the latter, through binding to and hydrolyzing ATP, provides transport energy and reverse concentration transfer for H+ outside cells, maintaining the normal range of intracellular pH value. A V1 domain includes three A subunits and three B subunits^21, 22^. The *ATP6V1A* gene encodes the A subunit in the structural domain V1 of V-ATPases on the membrane of lysosomes, which is important for the maintenance of pH values on both sides of the lysosomal membrane, and for the ensurance of the normal functions of lysosomes, autolysosomes and lysosomal proteolytic enzymes^23^.

Our previous studies^24–26^ found that pre-treatment with proton pump inhibitors (PPIs) can significantly inhibit the expression of V-ATPase in GC cell line SGC7901, and reverse multidrug resistance in GC through the down-regulation of PI3K/AKT/mTOR signaling pathway. PPIs mainly act on the H+/K+-ATP enzyme of gastric parietal cells. It is also found that, in addition to acting on gastric parietal cells, some V-ATPases of other types of cells also appear to be susceptible to inhibition by same inhibitors^27^. Based on these observations, we previously proposed that PPIs might affect the transcription of the *ATP6V1A* gene, thereby influencing the proton pump function with subsequent consequences including inhibition of proteolytic enzymes in lysosomes and interference of the autophagy process.

Given that the regulation of the *ATP6V1A* gene by both endogenous and exogenous factors is largely unknown, this study set to address the transcriptional regulation of this gene in two human GC cell lines. We first functionally cloned the promoter region of the *ATP6V1A* gene and studied its binding domains and interaction with the transcription factor Yin Yang 1 (YY1) that possesses multiple functions in a variety of biological processes as well as in the occurrence and development of tumors. In addition, YY1 has been reported to be upregulated in human cervical carcinomas and may serve as a potential therapeutic target for the treatment of HPV-positive cervical cancer^28^. RNAi-mediated knockdown and over-expression of YY1 in HGC-27 and AGS gastric cancer cells in a reporter gene system led to corresponding changes in *ATP6V1A* mRNA and protein expression. Thus, we conclude that the expression of *ATP6V1A in* human GC cells is positively regulated by YY1.

## Results

### Expression level of the *ATP6V1A* gene is elevated in gastric cancer patients

**Figure 1:**
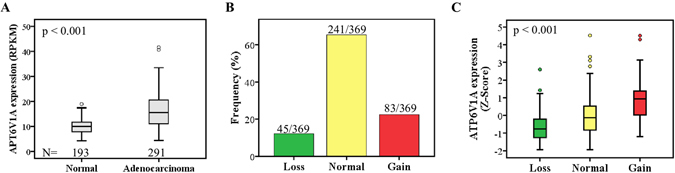
*ATP6V1A* gene is altered in gastric cancer patients. (**A**) Expression level of *ATP6V1A* is significantly elevated in GC in comparison to normal stomach tissues. (**B**) Frequency of DNA copy number alterations encompassing *ATP6V1A* gene in GC using TCGA dataset. (**C**) A significantly positive correlation was observed between DNA copy number and gene expression of *ATP6V1A* in TCGA dataset.

We first sought to investigate whether the *ATP6V1A* expression is altered in GC. The expression levels of *ATP6V1A* by RNA-sequencing in normal stomach and GC tissues were downloaded from the Genotype-Tissue Expression (GTEx) (http://www.gtexportal.org/home/) and The Cancer Genome Atlas (TCGA) (https://gdc-portal.nci.nih.gov/projects/TCGA-STAD), respectively. We found that the expression level of the *ATP6V1A* gene is significantly elevated in gastric adenocarcinomas in comparison to normal stomach tissues (Fig. 1A).

### DNA copy number increase is one of potential mechanisms for increased expression of *ATP6V1A* in gastric cancers

TCGA data were analyzed to search for the possible mechanisms underlying the upregulation of the *ATP6V1A* gene in the GC patients. Whereas mutations in *ATP6V1A* gene were relatively rare (4/369 = 1.08%), DNA copy number alterations encompassing *ATP6V1A* gene were frequently observed in GC (Fig. 1B). We then used a rank-based nonparametric test to determine whether the transcriptional expression levels are significantly associated with their copy number, and observed a significantly positive correlation between DNA copy number and gene expression (Fig. 1C).

### Functional cloning and characterization of human *ATP6V1A* promoter

**Figure 2:**
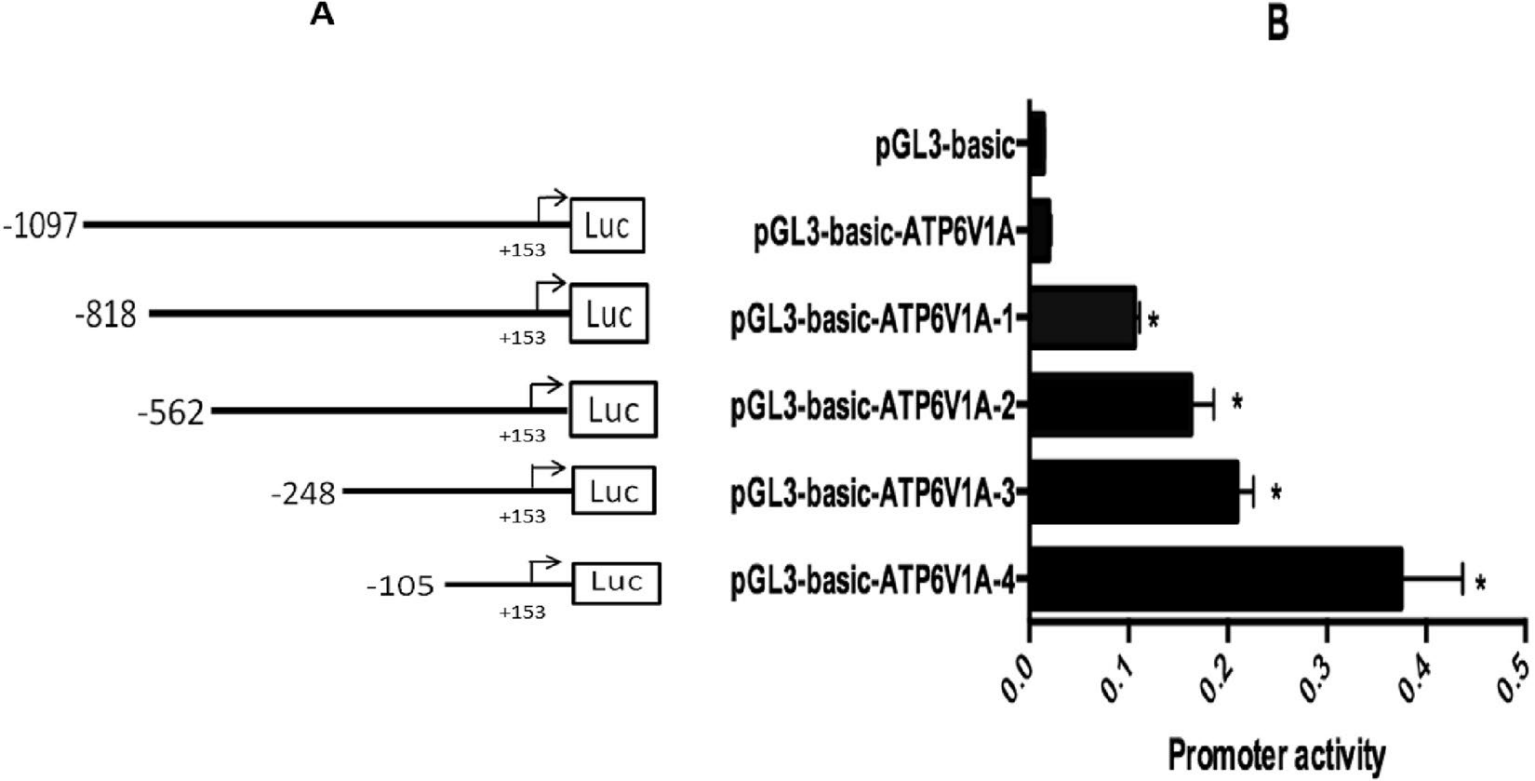
Deletion analysis of the human *ATP6V1A* gene promoter. (**A**) Schematic diagram of the *ATP6V1A* promoter constructs consisting of the 5′ flanking region with serial deletions cloned into the pGL3-basic vector. Arrow shows the direction of transcription. The numbers represent the end points of each construct. LUC: Luciferase vector. (**B**) The *ATP6V1A* gene promoter activity under various deletion constructs. The deletion plasmids were co-transfected with pGL3-basic expression vector into HGC-27 cells, which were harvested 24 h after transfection, and luciferase activity was measured in relative luciferase units (RLU). The values represent means ± SEM from three independent experiments (n = 3), *p < 0.001.

To further explore other mechanisms by which *ATP6V1A* overexpressed in GC, such as transcriptional regulation of *ATP6V1A* gene, we first cloned the promoter region (from −1097 to +153 bp) of human *ATP6V1A* into the pGL3-basic vector to yield plasmid pGL3-basic-ATP6V1A (Fig. 2A). Then, HGC-27 gastric cancer cells were transfected with plasmid pGL3-basic-ATP6V1A, and the corresponding luciferase activity was assessed in the dual luciferase reporter assay. Surprisingly, compared to the control cells transfected with plasmid pGL3-basic only, GC cells transfected with pGL3-basic-ATP6V1A showed no significant promoter activity (Fig. 2B), indicating that there might exist *cis*-acting elements covering the region from −1097 to +153 bp.

To further identify the active regions in the promoter of *ATP6V1A*, a series of luciferase reporter plasmids with truncated *ATP6V1A* promoters at different lengths were constructed (Fig. 2A). As shown in Fig. 2B, plasmid pGL3-basic-ATP6V1A-4 that contains the *ATP6V1A* promoter fragment −105 to +153 bp yielded the highest fluorescence activity compared to pGL3-basic-ATP6V1A and pGL3-basic (P < 0.001). This suggests that the most active region of the *ATP6V1A* promoter may be located at the region from −105 to +153 bp. In addition, in comparison with the control HGC-27 cells that were transfected with pGL3-basic, cells with pGL3-basic-ATP6V1A-1 (containing the promoter sequence of *ATP6V1A* from −818 to +153 bp), pGL3-basic-ATP6V1A-2 (containing the promoter sequence of *ATP6V1A* from −562 to +153 bp) and pGL3-basic-ATP6V1A-3 (containing the promoter sequence of *ATP6V1A* from −248 to +153 bp) all showed significant fluorescence activities (Fig. 2B), indicating that these promoter fragments also had strong promoter activities in the reporter system and the functional promoter region of the *ATP6V1A* gene is present in the sequence from *ATP6V1A* −818 to +153 bp.

### Identification of YY1 binding sites in human *ATP6V1A* gene promoter

**Figure 3:**
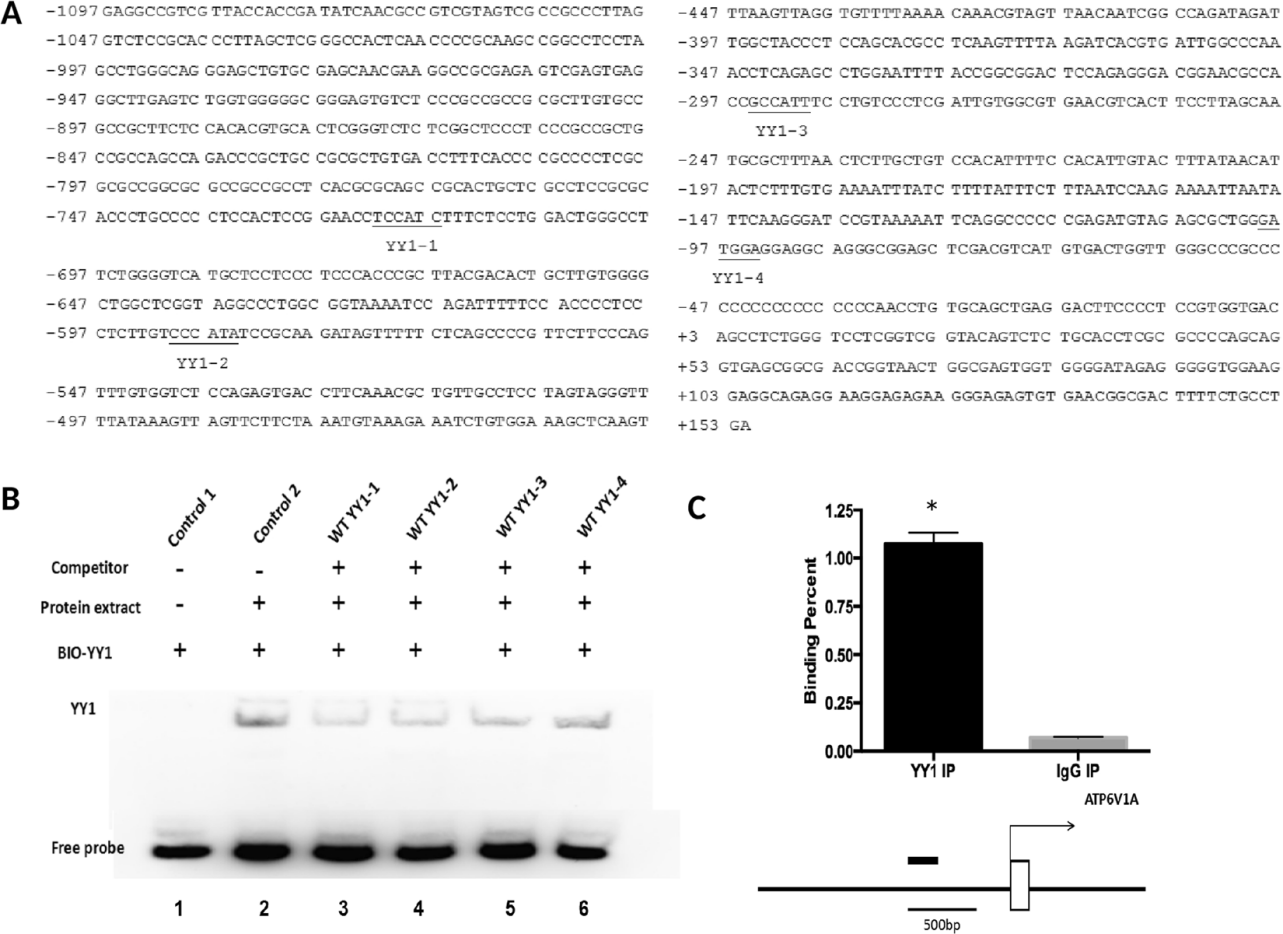
Identification of the YY1 binding sites in the *ATP6V1A* promoter. (**A**) The nucleotide sequence of the human *ATP6V1A* gene from −1097 to +13 bp. The putative transcription factor YY binding sites are underlined. (**B**) Excessive unlabeled oligonucleotide with the same sequence as the probe effectively competed with the probe binding by the YY1 protein and decreased its binding affinity, at a concentration of 50 times higher than the probe. In the 4 putatively predicted YY1 binding sites within the *ATP6V1A* promoter region, YY1-1, YY1-2 and YY1-3 oligonucleotides competed with the probe binding with YY1 at a concentration of 25 times higher to the probe, thereby significantly reducing the band intensity of DNA-protein complex of the probe with the YY1 protein in the lysate. (**C**) Chromatin immunoprecipitation (ChIP) assay for the YY1 binding to the *ATP6V1A* promoter. Left panel: Products of YY1 could be amplified in the co-immunoprecipitation with an anti-YY1 antibody, but not in the negative control samples containing a normal IgG, both of which were statistically different. Right panel: Schematic presentation of the region relative to the *ATP6V1A* transcriptional start site used as primer to test YY1 specific binding.

A computer-based analysis of transcription factor binding sites showed that a total of four putative YY1 binding sites were located in the 1251 bp promoter region of human *ATP6V1A* gene (Fig. 3A). An electrophoretic gel mobility shift assay (EMSA) was employed to verify whether these YY1 binding sites are functional or not. A 27 bp biotin-labeled double-stranded oligonucleotide probe, Bio-YY1, was synthesized and utilized for this assay. After incubation for up to 40 min at room temperature in dark, the biotin-oligonucleotide probe could specifically form a DNA-protein complex with the nucleoprotein sample, being presented as a shifted band in the EMSA gel (Fig. 3B).

To test the specificity of the YY1 binding to the promoter region of *ATP6V1A*, we carried out competition binding assay. As shown in Fig. 3B, excessive unlabeled oligonucleotide with the same sequence could effectively compete with the probe binding by the YY1 protein, and could evidently decrease the binding affinity of the probe to YY1 at a concentration of 50 times higher than the probe. In the four putatively predicted YY1 binding sites within the promoter region of the *ATP6V1A* gene, YY1-1, YY1-2 and YY1-3 oligonucleotides could compete with the probe in binding by the YY1 protein at a concentration of 25 times higher relative to the probe, thereby significantly reduce the band intensity of DNA-protein complex from the binding of the probe with the YY1 protein in the lysate. These findings suggest that the binding sites of YY1 protein in the promoter region of the *ATP6V1A* gene could be competitively bond by the YY1 protein (Fig. 3B).

To further validate the interaction of YY1 with the binding sites of YY1 in the *ATP6V1A* promoter region, the ChIP-QPCR method was employed. Equal amounts of normal IgG to an anti-YY1 antibody was added to serve as the negative control of co-immunoprecipitation. Products of YY1 could be amplified in the co-immunoprecipitation with the anti-YY1 antibody, whereas the target band could not be amplified from the YY1 primer in the negative control samples containing the normal IgG (Fig. 3C).

### YY1 transcriptionally regulates *ATP6V1A*

**Figure 4:**
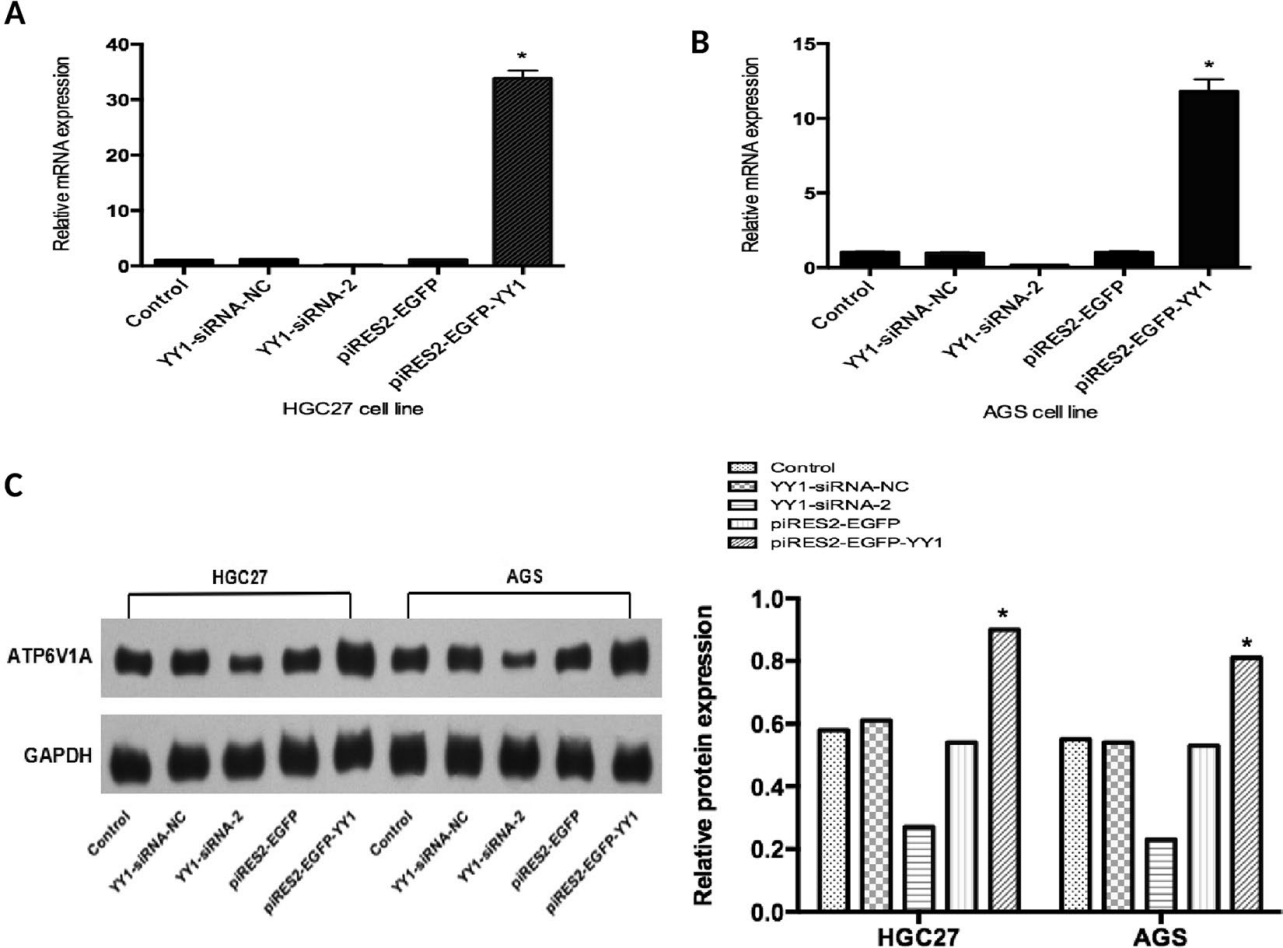
YY1 transcriptionally regulates ATP6V1A in GC cells. (**A**) RT-PCR for the effects of YY1 expression on *ATP6V1A* mRNA levels in GC cell lines. Following the transfection of YY1 siRNA into the HGC-27 gastric cancer cells, *ATP6V1A* mRNA levels were significantly decreased. In contrast, the mRNA expression levels of *ATP6V1A* were dramatically increased with the overexpression plasmid piRES2-EGFP-YY1. (**B**) Similar results were also detected in the AGS gastric cancer cells. (**C**) Western blot for the effects of YY1 expression on ATP6V1A protein levels in GC cell lines. Left panel: Western blot showing that *ATP6V1A* protein levels were significantly decreased after transfection of YY1 siRNA into the HGC-27 and AGS gastric cancer cells, whereas the protein expression levels of *ATP6V1A* were significantly increased when these cells were transfected with the overexpression plasmid piRES2-EGFP-YY1. Note that GAPDH was used as a loading control. Right panel: Quantitative analysis of the band intensity from (**A**). *p < 0.05.

To further investigate whether YY1 transcriptionally regulates *ATP6V1A*, both HGC-27 and AGS GC cells were transferred with the constructed YY1 siRNA for knockdown and plasmid piRES2-EGFP-YY1 for overexpression, respectively. YY1-siRNA-NC and piRES2-EGFP were respectively used as a control. The mRNA and protein expression levels of *ATP6V1A* were assessed by qRT-PCR and Western blot assay, respectively. Our results showed that, following the transfection of YY1 siRNA into the HGC-27 gastric cancer cells, *ATP6V1A* mRNA and protein levels were significantly decreased (Fig. 4). In contrast, the mRNA and protein expression levels of *ATP6V1A* were dramatically increased when these cells were transferred with the overexpression plasmid piRES2-EGFP-YY1. Similar results were also detected in the AGS gastric cancer cells (Fig. 4). Taken together, these results indicated that YY1 may play an important regulatory role in the transcription of *ATP6V1A* in gastric cancer cells.

**Figure 5:**
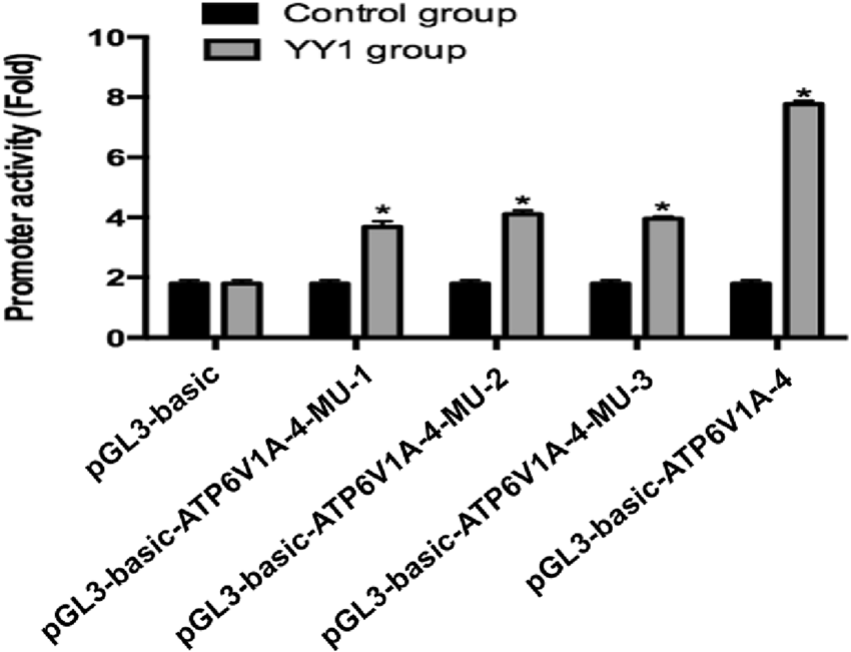
YY1 increases ATP6V1A transcriptional activity in GC cells. Plasmid piRES2-EGFP-YY1 was co-transfected into HGC27 GC cells with a plasmid containing pGL3-basic-ATP6V1A-4 or its mutant. In comparison with the negative control transfected with the plasmid pGL3-basic, the *ATP6V1A* promoter activity was significantly increased after transfected with piRES2-EGFP-YY1 with pGL3-basic-ATP6V1A-4, pGL3-basic-ATP6V1A-4-MU-1, pGL3-basic-ATP6V1A-4-MU-2 and pGL3-basic-ATP6V1A-4-MU-3, respectively. Moreover, the *ATP6V1A* promoter activity was much higher in those cells transfected with the plasmid pGL3-basic-ATP6V1A-4 harboring the complete promoter fragment than those plasmids with deletion constructs, including pGL3-basic-ATP6V1A-4-MU-1, pGL3-basic-ATP6V1A-4-MU-2 and pGL3-basic-ATP6V1A-4-MU-3. The values represent means ± SEM. n = 3, *p < 0.05.

To further determine whether the expression of YY1 affects the increased activity of various *ATP6V1A* promoters with different lengths, overexpression plasmid piRES2-EGFP-YY1 were co-transfected into HGC27 GC cells with pGL3-basic-ATP6V1A-4, pGL3-basic-ATP6V1A-4-MU-1, pGL3-basic-ATP6V1A-4-MU-2, pGL3-basic-ATP6V1A-4-MU-3, and pGL3-basic, respectively. The activity of the promoter was examined 24 h after transfection. Compared with the negative control transfected with the plasmid pGL3-basic, the *ATP6V1A* promoter activity was significantly increased after transfected with plasmids pGL3-basic-ATP6V1A-4, pGL3-basic-ATP6V1A-4-MU-1, pGL3-basic-ATP6V1A-4-MU-2 and pGL3-basic-ATP6V1A-4-MU-3, respectively (Fig. 5). Moreover, the *ATP6V1A* promoter activity was much higher in those cells transfected with the plasmid pGL3-basic-ATP6V1A-4 harboring the complete promoter fragment than those plasmids with a promoter containing a deletion, including pGL3-basic-ATP6V1A-4-MU-1, pGL3-basic-ATP6V1A-4-MU-2 and pGL3-basic-ATP6V1A-4-MU-3 (Fig. 5).

### Expression level of *ATP6V1A* is positively correlated with expression level of *YY1* in gastric cancer

**Figure 6:**
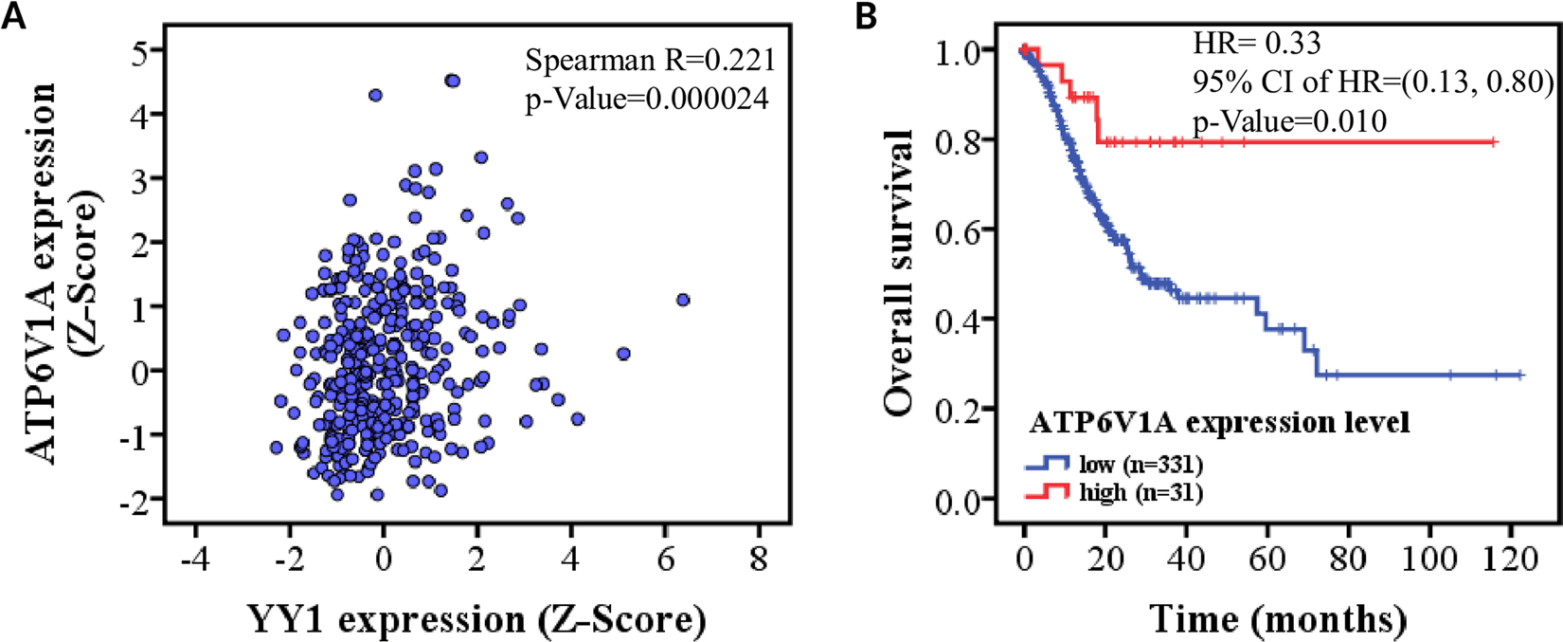
The correlation of *ATP6V1A* expression with *YY1* and prognosis in gastric cancer. (**A**) The expression levels of *ATP6V1A* are positively correlated with those of YY1 in gastric cancer. Through analysis of *YY1* and *ATP6V1A* mRNA expression in GC tissues of 369 individuals in the TCGA dataset, a significant correlation (Spearman correlation coefficient R = 0.221, P = 0.000024) was observed. (**B**) The prognostic value of *ATP6V1A* expression for GC patients was shown with the Kaplan-Meier analysis of the TCGA dataset, which indicated that a high level of *ATP6V1A* favors good prognosis in GC patients.

Through the analysis of YY1 and *ATP6V1A* mRNA expression in GC tissues of 369 individuals in the TCGA dataset, a significant correlation (correlation coefficient R = 0.218, P = 0.000024) was observed (Fig. 6A), which further confirmed that YY1 transcriptionally regulates the expression of *ATP6V1A*.

### Higher expression level of *ATP6V1A* favors a good prognosis in gastric cancer

To further assess the importance of *ATP6V1A* gene in GC development, we evaluated its prognostic value for GC patients in TCGA dataset using Kaplan-Meier analysis. The patients were stratified based on the *ATP6V1A* expression Z-Score. If the *ATP6V1A* expression Z-Score >1.70 (right tail bound of normal distribution at 0.95), we defined the patients with high expression of *ATP6V1A*. The remaining patients were defined with low expression of *ATP6V1A*. We found that high level of *ATP6V1A* favors good prognosis inpatients (Fig. 6B).

## Discussion

As a special proton pump of certain types of mammalian cells, V-ATPases have an important function of maintaining relatively neutral intracellular pH, acidic luminal pH, and acidic extracellular pH through pumping protons into the relative microenvironments^29^. Previous studies have reported that V-ATPases are overexpressed in many human malignant tumors, including gastric cancer^30, 31^. For example, immunohistochemical studies have identified the overexpression of V-ATPases in breast and lung cancer^32, 33^. In this study, we found that *ATP6V1A* overexpressed in GC by analyzing the gene expression in cells.

In this study, we sought to identify the molecular mechanisms by which *ATP6V1A* expression is elevated in GC. Changes in DNA copy number are often observed in tumors^34, 35^, and DNA copy number aberrations are one of the mechanisms that can result in a change in gene expression in tumor progression^36, 37^. We demonstrated in this study that genomic DNA copy number increase of *ATP6V1A* is significantly correlated with its gene expression. To search for other possible mechanisms, we constructed a series of plasmids containing various truncated *ATP6V1A* promoter regions. Through the dual luciferase reporter assay, the −105~+153 bp region was identified as the one with the strongest promoter activity for the transcriptional activation of *ATP6V1A* gene. Based on computer sequence analysis, human *ATP6V1A* promoter region was predicted to contain putative binding sites for several transcription factors, including YY1, E2F-1 and HRE.

YY1 is a member of the GLI-Kruppel family of zinc finger transcription factors. It is a multifunctional protein that participates in the regulation of many normal physiological processes, such as growth, differentiation, replication and cell proliferation^38–40^. Due to its function in inhibiting and activating transcription of numerous mammalian gene promoters, it is called Yin Yang 1 transcription factor^41^. Increasing studies have indicated that it also plays an important role in the occurrence and development of tumors by regulating tumor-associated genes through interacting with different protein cofactors^42–44^. YY1 is known to interact with the tumor suppressor gene p53 that is mutated in more than 50% tumorigenesis. YY1 can inhibit p53 function through multiple mechanisms^45, 46^. Recently, it was reported that the promoter region of dystrophin protein 71 (Dp71) (the smallest encoding gene of Duchenne muscular dystrophy) in hepatocellular carcinoma cells contained a binding site for YY1, and a point mutation in this binding site can reduce the promoter activity of this gene significantly^47^.

The transcriptional regulation of YY1 in GC cells has not been reported thus far. The EMSA assay of YY1 suggested that among the four predicted binding sites for YY1 in the *ATP6V1A* promoter region, and in addition, the oligonucleotides YY1-1, YY1-2 and YY1-3 were able to compete with the DNA probe for YY1 protein binding at a concentration 50 times higher than that of the probe, confirming the specificity of the DNA-protein complex formed by the probe and YY1 protein. CHIP assay further proved the interaction between the YY1 protein and the above three binding sites. Moreover, an increase in *ATP6V1A* promoter activity could be observed after the YY1 overexpression plasmid was co-transfected with *ATP6V1A* promoter plasmid, as shown in Fig. 3.

In order to further study the influence of YY1 on the mRNA and protein levels of the *ATP6V1A* gene, we constructed YY1 siRNA and piRES2-EGFP-YY1 plasmids for YY1 knockdown and overexpression assays. Our data clearly revealed a corresponding decrease or increase in *ATP6V1A* expression. This demonstrated that YY1 can regulate the transcription of the *ATP6V1A* gene mediated by its binding to the promoter region.

Through Kaplan-Meier survival analysis on 376 gastric adenocarcinoma samples in the TCGA database, we found that a high expression level of the *ATP6V1A* gene suggests better prognosis, and the same result was also demonstrated by the Kaplan-Meier plotter (http://kmplot.com/analysis/index.php?p=service&cancer=gastric) (see Fig. 6B). Since no specific treatment information was detailed in the TCGA samples, we can only speculate that the reason for this is that a high expression level of *ATP6V1A* may lead to increased sensitivity of cancer patients to other therapeutic measures such as chemotherapy, thus improving the prognosis of cancer patients.

In summary, in this work, we revealed that YY1 plays an important regulatory role in the transcription of the human *ATP6V1A* gene. Understanding of such role of YY1 may provide new insights into the abnormal regulation of GC-associated genes and pathways in cancer cells. Further studies are warranted to determine YY1-mediated functional changes and its interaction with other related genes in the pathogenesis of GC.

## Methods

### Datasets used in this study

The data for expression of *ATP6V1A* by RNA-sequencing in normal stomach and GC tissues were obtained from both GTEx (http://www.gtexportal.org/home/) and TCGA (https://gdc-portal.nci.nih.gov/projects/TCGA-STAD), we renormalized data based on total reads for each sample to generate RPKM (Reads Per Kilobase of transcript per Million mapped reads) and then compare the expression of *ATP6V1A* between normal stomach and GC tissues. The information about copy number change, mutation, expression levels of *ATP6V1A* and *YY1* in the TCGA study were obtained from the cBioPortal for cancer genome (http://www.cbioportal.org/). The clinical information of TCGA patients was retrieved from the website (http://www.cbioportal.org/study?id=stad_tcga#clinical).

### Cell lines and cell culture

The human gastric adenocarcinoma cell lines HGC27 and AGS were cultured in RPMI-1640 (Hyclone, USA) supplemented with 10% fetal bovine serum (Hangzhou Sijiqing Biological Engineering Materials, China) and antibiotics (100 U/ml penicillin and 100 μg/ml streptomycin) in humidified air with 5% CO_2_ at 37 °C (Thermo Direct Heat CO_2_, USA).

### Transfection

The YY1-siRNA, pIRES2-EGFP-YY1 and respective negative controls were purchased from GenePharma (Shanghai, China). The specific sequences of YY1 siRNA were as follows: (F) 5′-CAUGUCCAUUAUUCGUGATT-3′(R) 5′-UCACGAAUGGCCAUGTG-3′. A non-coding siRNA (Invitrogen, Carlsbad, CA, USA) was used as a negative control. Transfection was performed using Lipofectamine 2000 (Invitrogen, Carlsbad, CA, USA) according to the manufacturer’s instructions. Briefly, after trypsinization, cells were resuspended in an antibiotic-free medium and mixed with Opti-MEM (Gibco, Carlsbad, CA, USA) including 25 nM siRNA and Lipofectamine 2000. After incubation for 20 min at room temperature, cells were diluted with culture medium and seeded into a 60-mm dish. For cell viability assays, siRNA-transfected cells were re-seeded into a 48-well plate at 48 h after transfection. The silencing efficiency of V-ATPase V1A subunit siRNA was measured by the expression of mRNA and protein.

### Real-time PCR

Total RNA was isolated from HGC27 and AGS cell lines using Trizol reagent (Invitrogen) according to the manufacturer’s instructions. Reverse transcription reactions were performed with Transcriptor First Strand cDNA Synthesis Kit (Roche, Indianapolis, IN, USA). PCR primers were designed using Premier Primer 5.0 software. Real-time PCR with SYBR Green PCR MasterMix (Applied Biosystems, Foster City, CA, USA) was performed using an ABI Prism 7500 Sequence Detector instrument (Applied Biosystems). Fluorescent signals were collected during extension phase. Ct values of the sample were calculated, and transcript levels were analyzed employing the 2−ΔΔ Ct method.

### Western blot analysis

Cellular proteins were extracted in lysis buffer. The proteins were then separated by SDS-PAGE and electrophoretically transferred onto polyvinylidene fluoride (PVDF) membranes. The membranes were then probed with a polyclonal mouse antibody against V-ATPase subunit V1A (Taiwan Abnova, China) overnight at at 4 °C followed by incubation with horseradish peroxidase-conjugated goat anti-mouse antibody (1:1000, KPL). The protein bands were detected by the ECL kit (KeyGEN Biotech, Nanjing, China).

### Dual luciferase reporter assay

First, the luciferase promoter reporter constructs of different lengths were created. Briefly, the promoter sequence of human *ATP6V1A* was generated by PCR and then inserted into the pGL3-basic expression vector. Targeted deletion of the full-length promoter was synthesized by KeyGEN Biotech (Nanjing, China). The final constructs were termed pGL3-basic-ATP6V1A and pGL3-basic-ATP6V1A-mut. These recombinant vector constructs were confirmed by restriction endonuclease digestion analysis and DNA sequencing. For dual luciferase reporter assays, the HGC-27 cells were cultured in 24-well plates and transfected with 100 ng of pGL3-basic-ATP6V1A, pGL3-basic-ATP6V1A-mutant, and pGL3-basic, respectively, using Lipofectamine 2000 (Invitrogen, USA). The cells were harvested after transfection for 48 h, and assayed using the Dual-Luciferase Reporter Assay kit (Promega, USA) according to the manufacturer’s instructions.

### Electrophoretic gel mobility shift assay (EMSA)

Cell lysates were extracted with RIPA DOC buffer (1% Triton X-100, 0.1% sodium dodecyl sulfate, 1% sodium deoxycholate, 0.15 M NaCl, 0.05 M Tris–HCl, pH 7.2, and 0.5 mM phenylmethylsulfonyl fluoride) and stored at −80°. EMSA was performed as described previously^48^, using the Light Shift chemiluminescent EMSA kit (Thermo Fisher Scientific, Wilmington, DE, USA). A nucleoprotein sample was employed that was from the cell lysate preparation. Biotin-labeled double-stranded DNA fragments and wild-type competitor oligonucleotides were acquired from Invitrogen (Thermo Fisher Scientific, Waltham, MA, USA) and utilized. These probes represent various *ATP6V1A* promoter regions, including positive control probe BIO-YY1, WT YY1-1, WT YY1-2, WT YY1-3 and WT YY1-4. The sequences of biotin-labeled double-stranded DNA probes and wild-type competitor oligonuleotides are listed in the supplement file (Table 1).

**Table 1:** The sequences of biotin-labeled DNA probes and wide-type competitors.

Bio-YY1	Bio-YY1-F	CGC TCC CCG GCC ATC TTG GCG GCT GGT
	Bio-YY1-R	ACCAGCCGCC AAGATGGCCG GGGAGCG
YY1-1	YY1-1-F	CGGAACCTCCATCTTTCTCC
	YY1-1-R	GGAGAAAGATGGAGGTTCCG
YY1-2	YY1-2-F	CTCTTGTCCCATATCCGCAA
	YY1-2-R	TTGCGGATATGGGACAAGAG
YY1-3	YY1-3-F	CGCCACCGCCATTTCCTGTC
	YY1-3-R	GACAGGAAATGGCGGTGGCG
YY1-4	YY1-4-F	TGCCTCCTCCATCCCAGCGC
	YY1-4-R	GCGCTGGGATGGAGGAGGCA

#### Chromatin immunoprecipitation assay (ChIP)

ChIP assays were performed as described previously^49^. Rabbit anti-YY1 antibody was purchased from Santa Cruz Biotechnology (Dallas, TX, USA). Protein-A/G agarose beads were obtained from Pierce (Rockford, IL, USA), and mouse IgG conjugated with magnetic beads, used as the negative control, was purchased from Cell Signaling Technology (Danvers, MA, USA). Besides the control IgG, the quantity of *ATP6V1A* DNA fragment precipitated and analyzed under the same conditions served as an additional control for the specificity of the binding between ChIP antibodies and their target genes. ChIP primers for the *ATP6V1A* promoter were synthesized by Invitrogen (Carlsbad, CA, USA) and listed as follows: (F) 5′-GTCCCATATCCGCAAGATAGTT-3′ (R) 5′-GAGCTTTCCACAGATTTCTTTAC-3′.

#### Statistical analysis

All experimental data are expressed as Means ± S.D. Statistical significance of differences of experiemntal data according to *YY1* expression was determined by Student’s t-test. The difference in expression of *ATP6V1A* among copy numbers of *ATP6V1A* in TCGA dataset was assessed by non-parametric Kruskal-Wallis test. Correlation between *YY1* and *ATP6V1A* expression in TCGA dataset was calculated using Spearman’s correlation. The prognostic significance of *ATP6V1A* with overall survival of GC patients was assessed using the Kaplan-Meier analysis. The patients were stratified based on the *ATP6V1A* expression Z-Score. The cutpoint of Z-Score is 1.70 (right tail bound of normal distribution at 0.95). All analyses were performed with SPSS 17.0 (SPSS Inc., Chicago, IL, USA) for Windows. The significance level was set at p < 0.05.
